# P-1198. Inner Zones of Inhibition in Disk Diffusion Testing of Cefiderocol Best Reflect Activity Against Acinetobacter baumannii

**DOI:** 10.1093/ofid/ofaf695.1391

**Published:** 2026-01-11

**Authors:** Naoki Kohira, Dai Miyagawa, Hidenori Yamashiro, Motoyasu Onishi, Christopher M Longshaw, Boudewijn L DeJonge, Yoshinori Yamano

**Affiliations:** Shionogi & Co., Ltd., Toyonaka, Osaka, Japan; Shionogi & Co., Ltd., Toyonaka, Osaka, Japan; Shionogi & Co., Ltd., Toyonaka, Osaka, Japan; Shionogi & Co., Ltd., Toyonaka, Osaka, Japan; Shionogi B.V., London, England, United Kingdom; Shionogi Inc., Florham Park, NJ; Shionogi & Co., Ltd., Toyonaka, Osaka, Japan

## Abstract

**Background:**

Cefiderocol (FDC) is a siderophore-conjugated cephalosporin with activity against aerobic Gram-negative bacteria, including multidrug-resistant isolates. The disk diffusion (DD) method for FDC was approved by the Clinical and Laboratory Standards Institute (CLSI), but non-reproducible appearance of colonies within the zone of growth inhibition, especially observed with *Acinetobacter baumannii,* can complicate endpoint reading (Fig. 1). Here we investigated if reading outer zone of growth inhibition, which is more reproducible, can be used as endpoint reading.

**Figure 1.**
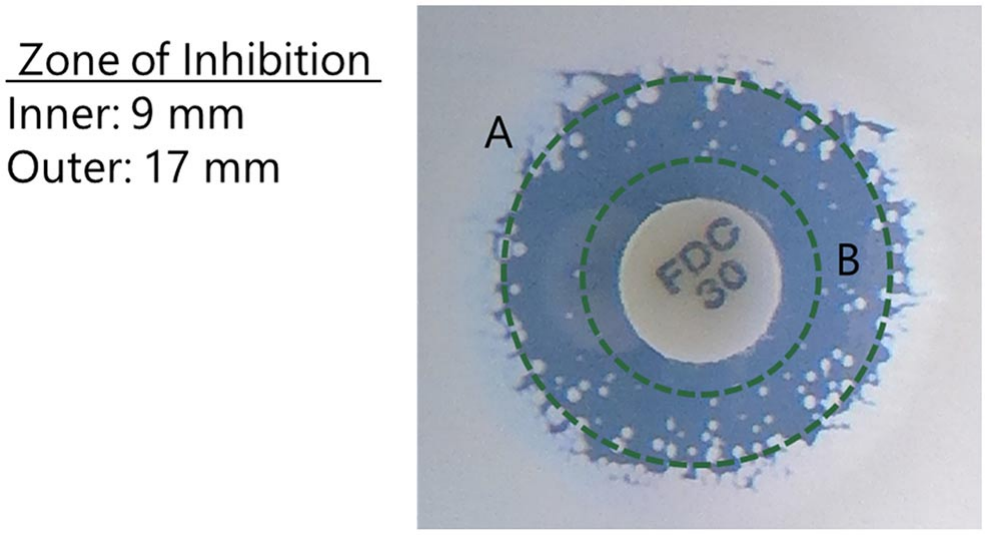
Example of colonies within predominant zone of growth inhibition. A: Predominant inhibition zone (outer inhibition zone). B: Inner zone of inhibition taking colonies within zone of inhibition into account.

**Figure 2.**
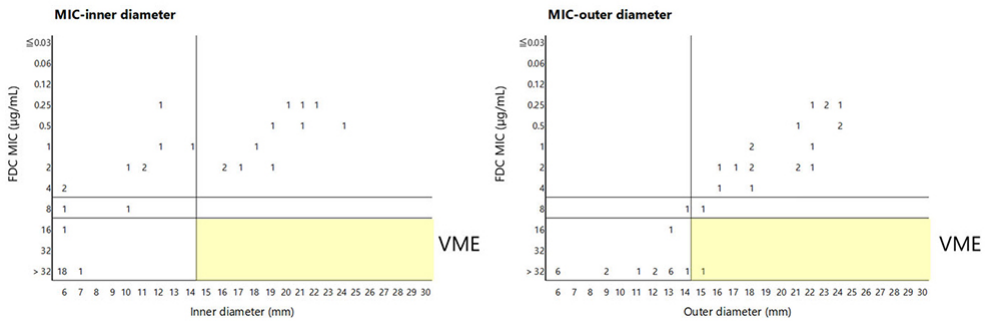
Correlation of inhibition zone diameters obtained by DD and MIC of FDC determined with broth microdilution. Modal MIC (n=9 - 30) and mean diameter (n=9) were calculated.

**Methods:**

41 *A. baumannii* strains were used for this study. Seventeen of 41 *A. baumannii* strains showed colonies in disk inhibition zone. MIC measurements in iron-depleted cation-adjusted Mueller Hinton broth (Becton Dickinson BBL) and DD testing using Mueller-Hinton agar (Becton Dickinson) and FDC disk (Mast Group) were performed and interpreted according to CLSI protocols. *In vivo* efficacy was evaluated in a neutropenic murine thigh infection model using human-simulated regimen dosing of FDC ^1, 2, 3^.

**Figure 3.**
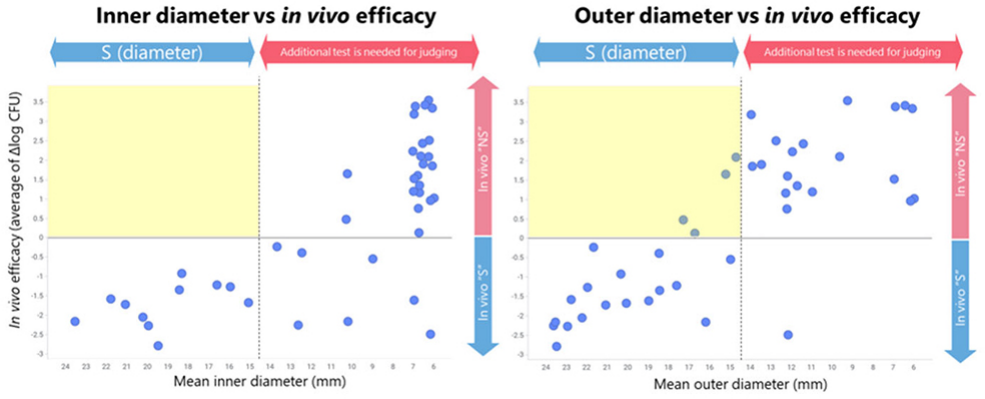
Correlation of mean inhibition zone diameters of DD test and in vivo efficacy. “S” indicates susceptible. Mean zone diameter was calculated by using 9 diameter data for each strain.

**Results:**

In correlation analysis between DD zone of growth inhibition and FDC MIC, inner diameters and MIC showed no very major error (VME), while outer diameters and MIC showed 2% (1/41) VME (Fig. 2). In correlation analysis between DD zone of growth inhibition and *in vivo* efficacy, all strains judged susceptible by inner diameter read-out showed a reduction in bacterial burden *in vivo,* whereas 19% (4/41) of strains judged susceptible by outer diameter read-out showed an increase in bacterial burden *in vivo* (Fig. 3).

**Conclusion:**

Using the inner diameter as read-out for zone of growth inhibition showed a better correlation with MIC and efficacy *in vivo*, and therefore use of inner diameter for judging susceptibility in *A. baumannii* using DD is suggested.

References

1. Monogue ML, *et al. Antimicrob Agents Chemother.* 2017;61(11):e01022-17

2. Gill CM, *et al*, *JAC Antimicrob Resist*. 2022;4(3):dlac047

3. Gill CM, *et al*, *J Antimicrob Chemother.* 2023;78(4):983-990

**Disclosures:**

Hidenori Yamashiro, Shionogi HQ: Employee Christopher M. Longshaw, PhD, Shionogi BV: Employee Boudewijn L. DeJonge, PhD, Shionogi Inc.: Employee Yoshinori Yamano, PhD, Shionogi HQ: Employee

